# Self-powered technologies for smart bearings

**DOI:** 10.1016/j.fmre.2025.04.007

**Published:** 2025-04-19

**Authors:** Likun Gong, Zhi Zhang, Ruifei Luan, Yuan Feng, Mang Gao, Chi Zhang

**Affiliations:** a CAS Center for Excellence in Nanoscience, Beijing Key Laboratory of Micro-nano Energy and Sensor, Beijing Institute of Nanoenergy and Nanosystems, Chinese Academy of Sciences, Beijing 101400, China; b School of Nanoscience and Engineering, University of Chinese Academy of Sciences, Beijing 100049, China; c Center on Nanoenergy Research, Institute of Science and Technology for Carbon Peak & Neutrality, Key Laboratory of Blue Energy and Systems Integration (Guangxi University), Education Department of Guangxi Zhuang Autonomous Region, School of Physical Science & Technology, Guangxi University, Nanning 530004, China; d Hunan Key Laboratory of Nanophotonics and Devices, School of Physics, Central South University, Changsha 410083, China

**Keywords:** Smart bearings, Self-powered, Triboelectric, Piezoelectric, Tribovoltaic effect

## Abstract

As industrial equipment becomes increasingly complex and efficient, smart bearings play a crucial role in monitoring the operating status of mechanical devices, improving efficiency, and extending service life. To achieve long-term stable operation of smart bearings and avoid the issues associated with replacing and maintaining traditional batteries, self-powered technologies have become a research hotspot in recent years. Self-powered smart bearings collect mechanical energy, vibrational energy, thermal energy, and other forms of energy from the surrounding environment and convert them into electrical energy, driving embedded sensors or functioning as self-powered sensors themselves. This enables real-time monitoring of bearing conditions and data transmission. Although significant progress has been made in self-powered technologies, comprehensive reviews that summarize self-powered technologies in the field of smart bearings and provide guidance for future development are still scarce. To fill this gap, this review summarizes five main energy harvesting methods for self-powered smart bearings: electromagnetic generation, piezoelectric generation, thermoelectric generation, triboelectric generation, and tribovoltaic generation technologies. By analyzing the principles, structures, and performances of each technology, this paper explores their applicability and potential in smart bearings and forecasts the future development direction of self-powered technologies for smart bearings. This review provides a reference for the design and application of smart bearings and promotes the widespread use of self-powered smart bearings in industrial equipment health management.

## 1. Introduction

### 1.1. Development of self-powered smart bearings

As a core component in rotating machinery, bearings are widely used in various critical equipment such as high-speed trains, wind turbines, and aircraft engines [1,2]. In these devices, the performance of bearings directly impacts the efficiency, reliability, and service life of mechanical systems [3,4]. Therefore, real-time monitoring of bearing operating conditions and timely detection of potential faults are crucial for avoiding equipment downtime, extending service life, and improving production safety [5,6]. However, during long-term operation, bearings often face problems such as wear, corrosion, electrical erosion, plastic deformation, and fracture, leading to failures. Particularly when working under high load, high speed, or extreme environments, bearing failure can cause significant damage to mechanical systems, even leading to more serious equipment breakdowns or personal injuries [7,8].

Traditional bearing fault diagnosis techniques rely on external signals such as vibration, temperature, and noise. However, these signals are often interfered with by noise and cannot accurately reflect the internal state of the bearing [[9], [10], [11]]. Therefore, the research on embedded smart bearings has gradually become an important approach to solving this issue. Against the backdrop of the Industrial Internet of Things (IIoT) and smart manufacturing, there is a growing demand for smart bearings with advanced functionalities. Smart bearings, by integrating miniature sensors, signal processing circuits, and wireless communication modules, can monitor the operating status of bearings in real time, detect potential faults, and transmit data remotely to cloud platforms for analysis and processing. The core goal of smart bearings is to predict and diagnose potential failures through accurate data analysis, enabling maintenance or replacement before failure occurs, thus avoiding unexpected downtime and severe damage to equipment [12,13].

With the rapid development of industrial IoT technology, smart bearings not only possess online monitoring and fault diagnosis functions but can also achieve remote data transmission and cloud computing analysis. However, the widespread application of smart bearings faces a major technical challenge: how to provide stable and long-lasting power supply to these sensors, signal processing, and wireless communication modules [[14], [15], [16]]. Traditional smart bearings typically rely on batteries as their power source. However, due to the limited battery life and the need for regular replacement and maintenance, this method is difficult to meet the requirements for long-term and stable operation. Especially in remote areas, harsh environments, or situations where equipment cannot be frequently shut down, battery replacement and maintenance become impractical. Moreover, the disposal of used batteries may also cause environmental pollution.

Therefore, the development of self-powered smart bearing technology has become the key solution to this problem. Self-powered technology supplies power to smart bearings by harvesting energy from the environment, eliminating the dependence on batteries and enabling long-term stable operation [[17], [18], [19]]. The basic structure of a self-powered smart bearing includes an energy harvesting module, a sensor module, a wireless transmission module, and data processing module. Typically, there are two technical routes: one is to harvest friction energy, thermal energy, and vibrational energy generated during bearing rotation and convert it into electrical energy, using the electrical signals to sense parameters such as speed and wear status, and process the signals for fault diagnosis [20]. The other route is to harvest energy during the bearing's rotation and convert it into electrical energy to provide continuous power supply for the system's integrated sensors (e.g., acceleration, temperature) and wireless transmission modules [21]. With the continuous development of self-powered technology, integrated and adaptive smart bearings will become an important part of future industrial equipment health management (Fig. 1).

**Fig. 1:**
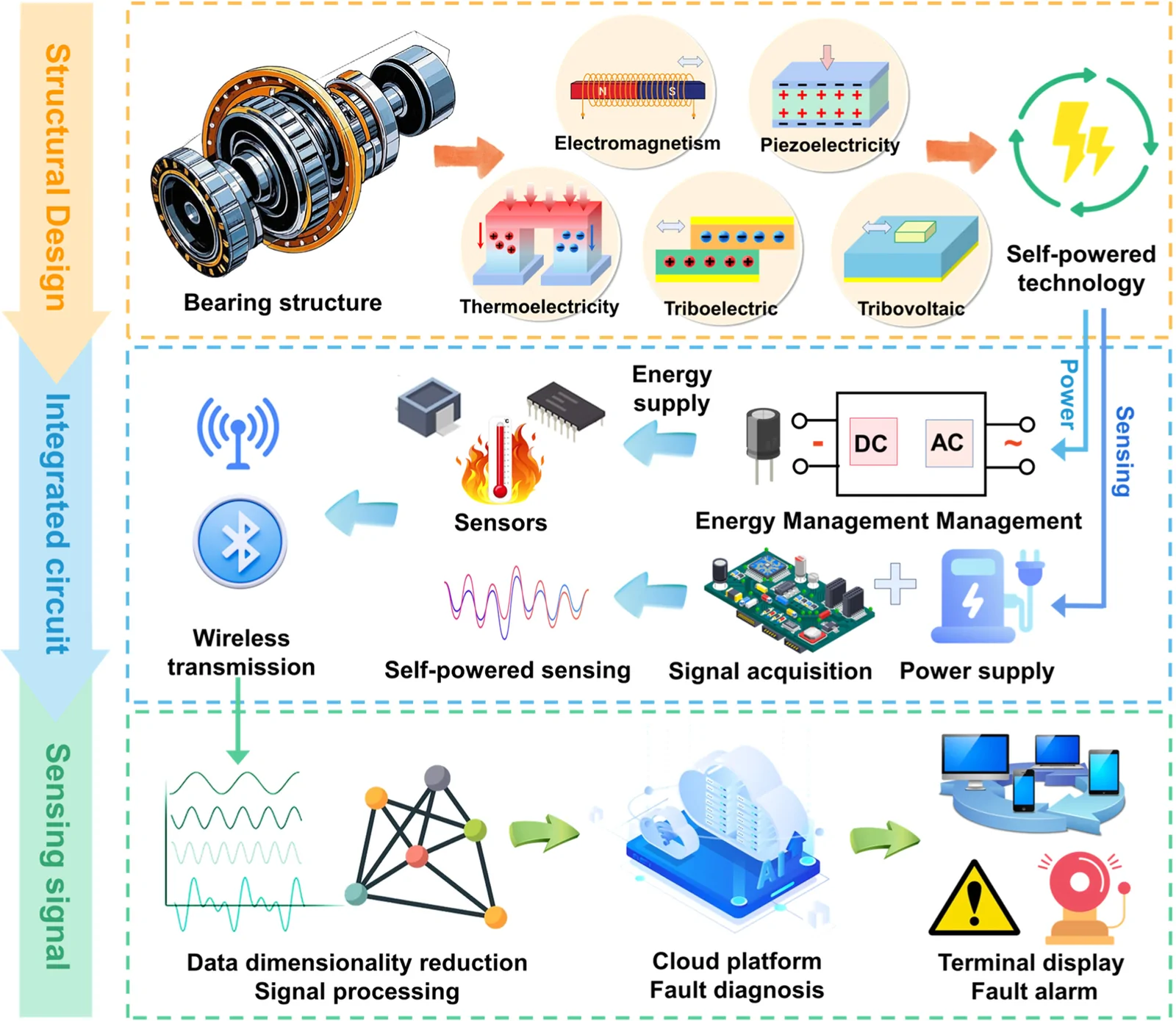
**Structure, function and application prospects of the self-powered smart bearing system**.

### 1.2. Introduction to self-powered technology

Currently, the main methods for self-powered smart bearings include electromagnetic, piezoelectric, thermoelectric, triboelectric, and tribovoltaic energy harvesting technologies. Each of these technologies has unique working principles and application advantages, enabling efficient and stable power supply according to different operating environments and requirements (Fig. 2).

**Fig. 2:**
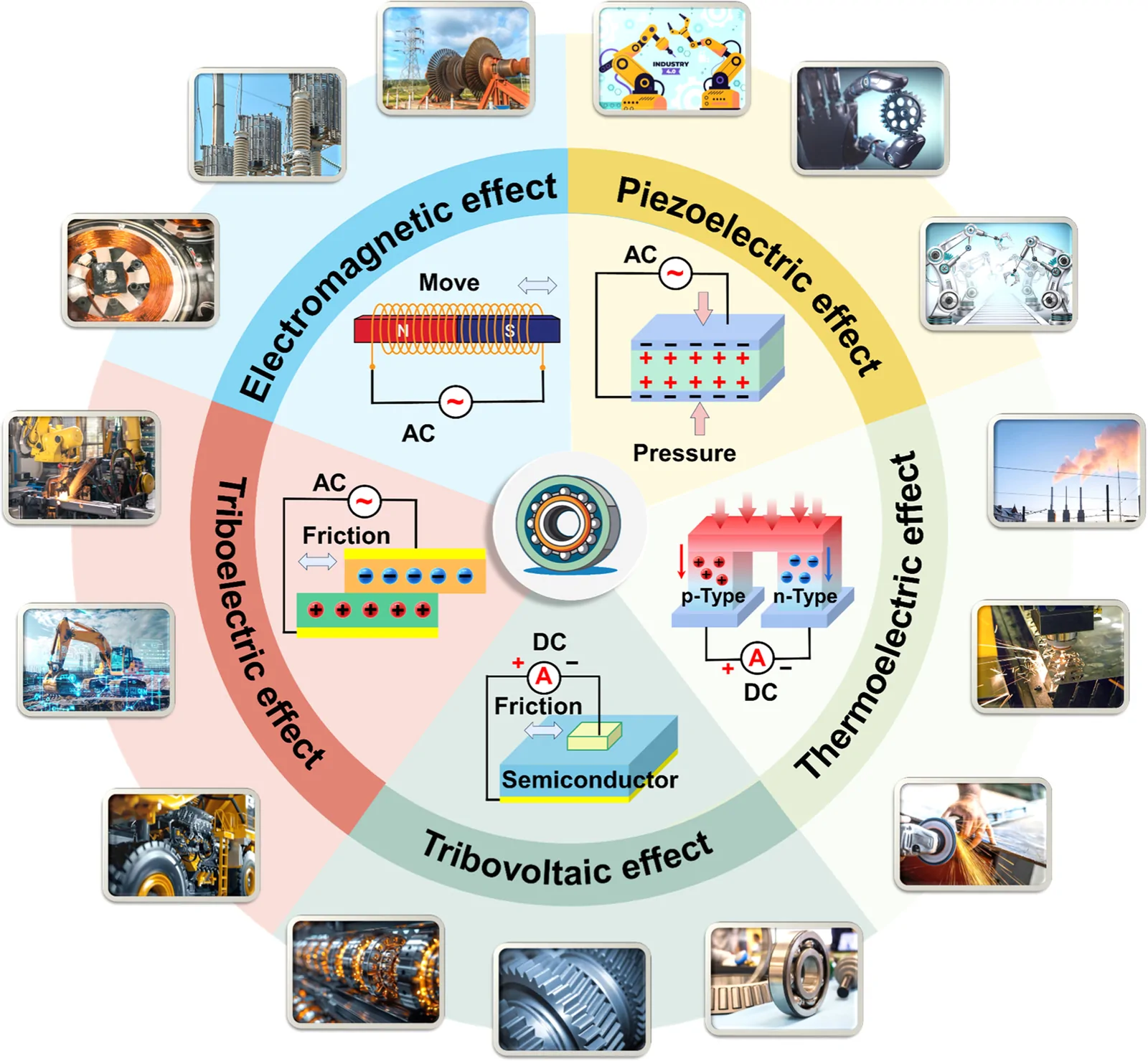
**Several common self-powered technologies of smart bearing**.

Electromagnetic generation technology is one of the earliest technologies widely applied to mechanical energy conversion. It is based on Faraday's law of electromagnetic induction, discovered in 1831, where an alternating current (AC) is generated in a conductor by changes in the magnetic field [22,23]. In smart bearings, electromagnetic generation typically uses the rotational motion of the bearing to drive relative movement between the rotor and stator, thereby generating electrical energy. Electromagnetic generators have good adaptability and can operate effectively at high frequencies, making them suitable for variable-speed or high-speed rotating devices (> 200 rpm). However, the size and structural design of electromagnetic generators are relatively complex, and their efficiency is limited in high-speed rotational applications [24,25].

Piezoelectric generation technology relies on the piezoelectric effect, where piezoelectric materials generate charges or voltage when subjected to external forces. This effect was first discovered in 1880 by French scientists Pierre and Jacques Curie [[26], [27], [28]]. Common piezoelectric materials used in smart bearings include lead titanate and zirconium titanate, which generate voltage when subjected to vibration or mechanical stress during operation. Piezoelectric generation technology has the advantages of a simple structure, rapid response, and high energy density, making it particularly suitable for vibration energy harvesting. However, its efficiency is relatively low in low-frequency vibrations or environments with small changes [29,30].

Thermoelectric generation technology converts temperature differences into electrical energy through the thermoelectric effect. The discovery of the thermoelectric effect dates back to 1834, where heat drives the movement of electrons in materials under different temperature conditions, generating direct current (DC) output [[31], [32], [33]]. In smart bearings, friction generates heat, providing an energy source for thermoelectric generation. Thermoelectric generation not only effectively utilizes excess heat generated during the operation of mechanical components but also helps dissipate heat, preventing overheating that could reduce bearing performance or shorten service life. However, the efficiency of thermoelectric generation is still limited by material properties, temperature differences, and equipment design [34,35].

Triboelectric generation technology, a recently emerging energy harvesting method, works on the coupling effect of contact electrification and electrostatic induction. The key to triboelectric generation is the contact and separation between the surfaces of two different materials, generating a potential difference through the transfer and distribution of electrons [36,37]. In 2012, Zhong Lin Wang's team first introduced the triboelectric nanogenerator, which has quickly become a popular energy harvesting method due to its simple structure, low cost, and high energy conversion efficiency [38]. Triboelectric generation is particularly suitable for harvesting mechanical energy from low-frequency vibrations and small movements and has been widely used in self-powered sensors and smart devices [[39], [40], [41]]. However, triboelectric nanogenerator-based self-powered bearings often require periodic structural designs, increasing complexity. Moreover, most triboelectric nanogenerators are based on polymer materials, which may degrade under the operating conditions of bearings [42].

Tribovoltaic generation technology is an advanced development of triboelectric generation, based on the tribovoltaic effect [43,44]. In 2020, Chi Zhang's team first defined and experimentally verified the tribovoltaic nanogenerator, which uses friction between metal-semiconductor or semiconductor-semiconductor interfaces to excite charge carriers, generating current [45]. During the friction process, the formation and breaking of atomic bonds at the interface release bond energy, which excites the charge carriers. Under the driving force of the built-in or interface electric field, the charge carriers move directionally, generating direct current [46]. The advantage of tribovoltaic generation technology lies in its ability to withstand high mechanical pressure and generate high electrical energy output even with small contact areas, making it highly suitable for bearing operating environments [47].

These five energy harvesting technologies have their own strengths and applications in smart bearings. In practical applications, selecting the appropriate energy harvesting technology based on different working environments and energy requirements is a critical issue in the design of self-powered smart bearings. Through reasonable system integration and optimization, energy harvesting efficiency can be effectively improved, ensuring the long-term stable operation of smart bearings. Although some reviews summarize recent self-powered methods, a comprehensive summary of self-powered technologies in the field of smart bearings is still lacking. Therefore, this review will provide a detailed discussion and analysis of the five main energy harvesting technologies for self-powered smart bearings and comprehensively evaluate their applications in smart bearings. We explore the working principles, features, advantages, and limitations of these technologies and perform a comparative analysis of their applicability in self-powered smart bearings. Finally, this paper discusses future trends and research directions in the development of smart bearings, aiming to provide theoretical support and technical reference for the design and application of smart bearings.

## 2. Electromagnetic power generation technology

As the most widely used power generation technology in the world today, electromagnetic power generation has played an important role in fields such as wind and hydroelectric power. It can produce stable electrical output over long periods, making it suitable for bearing monitoring systems that require a stable power supply. Zhang et al. [48] proposed an electromagnetic energy harvester based on a circular Halbach array. This design cleverly utilizes the characteristic of the Halbach array to enhance the magnetic field, optimizing the magnetic field distribution between the magnet and the coil to improve energy harvesting efficiency. The schematic diagram of the system shows a device composed of permanent magnets, coils, bearing end caps, cages, rollers, and an inner ring. The permanent magnets are arranged in a circular Halbach array in the bearing end cap, while the coils are fixed to the cage. As the bearing rotates, the relative motion between the end cap and the cage causes a change in the magnetic field, inducing a voltage in the coil (Fig. 3a). With a gap of 12 mm, the waveform of the induced voltage for different rotational speeds is presented. The experimental results match the simulation data, with the frequency and RMS value of the induced voltage showing the same trend of change with increasing speed, verifying that the proposed model can accurately predict the voltage response (Fig. 3b). The generator is made using 3D printing technology, with the rotor and stator made of PLA material and aluminum alloy, respectively. Over a speed range of 600 rpm to 1000 rpm, the system can produce voltages ranging from 2.79 V to 4.59 V and maximum average powers from 50.8 mW to 131.1 mW (Fig. 3c). Based on similar structures, Zhang et al. also validated the model's effectiveness under different system parameters through finite element simulations. Particularly when studying the effects of parameters such as the gap between the magnet and coil, speed, and coil radius on the output voltage, the experimental results demonstrated that the model is suitable for bearing condition monitoring applications (Fig. 3d) [49].

**Fig. 3:**
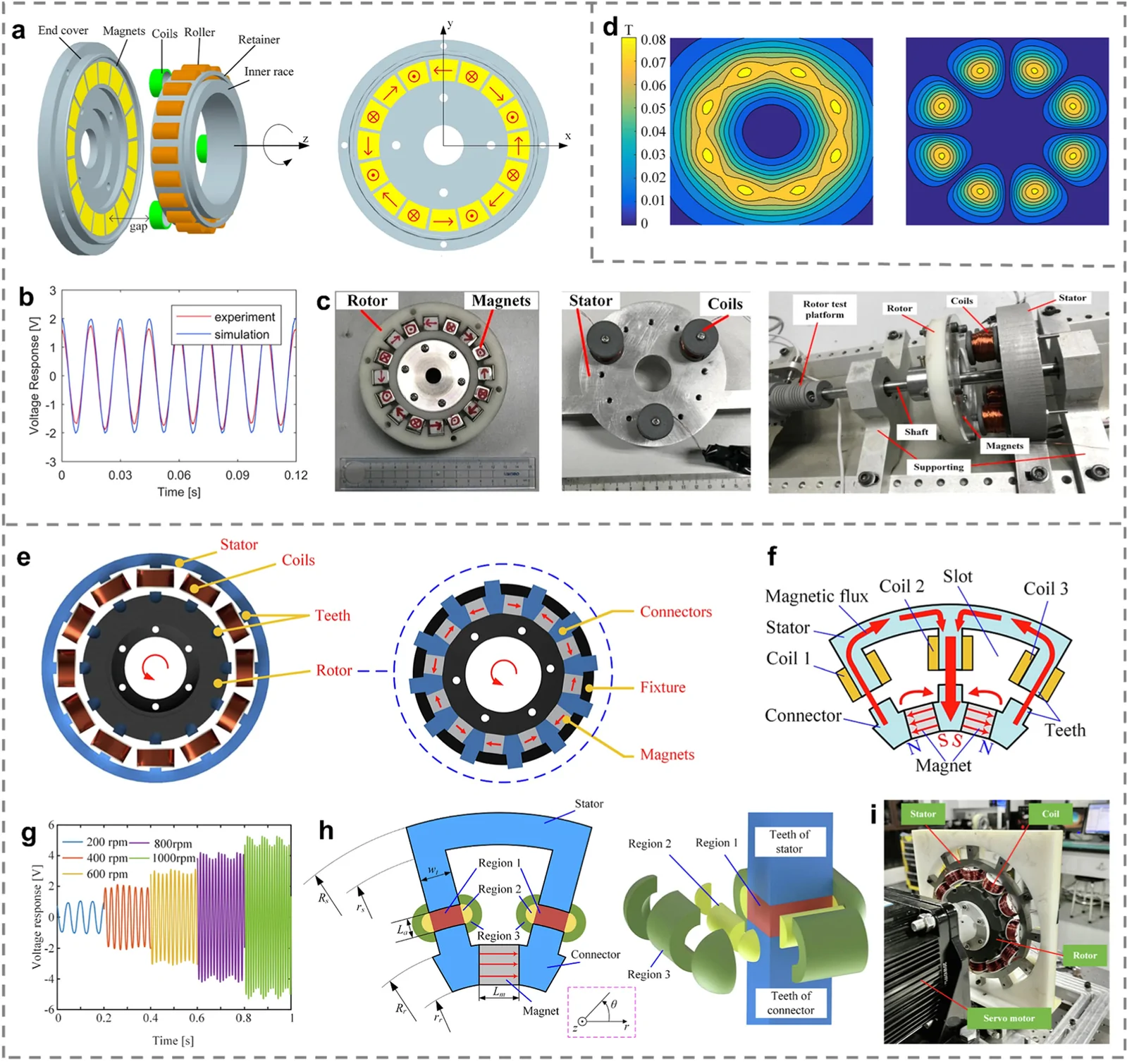
**Self-powered bearings based on electromagnetic power generation technology.** (a) Schematic of the electromagnetic energy harvester. (b) Simulation and experimental comparison of coil voltage responses at different rotational speeds. (c) Photo of the rotor with magnets and stator with coils [44]. (d) Magnetic flux density distribution [45]. (e) Schematic of the electromagnetic energy harvester structure. (f) Working principle of the electromagnetic energy harvester. (g) Voltage output of the energy harvester at different rotational speeds. (h) Magnetic field division schematic. (i) Physical image of the energy harvester [46].

In addition, Zhang et al. proposed an enhanced variable reluctance energy harvester (EVREH), designed specifically for self-powered bearing health monitoring under low-frequency rotational conditions [50]. This system uses periodic arrangements of magnets and teeth to increase energy harvesting performance by frequency up-conversion, allowing high energy density electrical output even in space-constrained environments. At a rotational speed of 200 rpm, EVREH can output a voltage of 8.7 V and a power of 726 mW, with a power density of 0.545 mW/(cm³·Hz²) (Fig. 3e). When the rotor teeth align with the stator, the magnetic flux changes, generating a voltage. As the number of rotor teeth increases, the frequency of magnetic flux variation rises, increasing the voltage output (Fig. 3f). At 1000 rpm, the maximum voltage response after connecting 12 coils in series reaches 43.1 V (Fig. 3g). To optimize the reluctance, they used a magnetic field division method to model the air-gap magnetic permeability and accurately calculate the magnetic flux distribution, further enhancing the model's accuracy (Fig. 3h). The experimental setup includes a servo motor, coils, connectors, and other components, with experimental data collected via an oscilloscope. This verified the EVREH's self-powering and condition-monitoring capabilities under low-frequency rotational conditions, providing stable electrical power support for wireless sensors in rotating equipment (Fig. 3i).

Gong et al. proposed an N-shaped electromagnetic energy harvester (NEMEH), designed to provide self-powering capabilities for wireless sensor nodes through the rotation of smart bearings [51]. This harvester combines a cylindrical Halbach array with N-shaped electrical steel embedded in the axle box bearing of high-speed trains. NEMEH enhances the magnetic performance and energy harvesting efficiency through the cooperation of arc magnets, N-shaped electrical steel, and coils, forming a concentrated magnetic flux closed loop (Fig. 4a). To optimize the design, Gong et al. derived a magnetoelectric coupling model and studied the effects of different structural parameters on the magnetic field and output response through theoretical modeling and finite element analysis (Fig. 4b). Over a speed range of 200 to 1200 r/min, NEMEH can output a maximum voltage of 14.6 V and a power of 4.26 W, demonstrating high energy harvesting capabilities. At 1200 r/min, the voltage waveform of the NEMEH prototype is highly consistent with theoretical predictions and simulation data, indicating that the system can stably output at different rotational speeds (Fig. 4c). The prototype's installation on the rotational test platform shows that the combination of the rotor and 36 arc-shaped permanent magnets with N-shaped electrical steel creates a powerful magnetic field. The experiments further verified that this harvester can provide sufficient power for wireless sensor nodes, proving its potential for self-powering and condition monitoring in smart bearings (Fig. 4d).

**Fig. 4:**
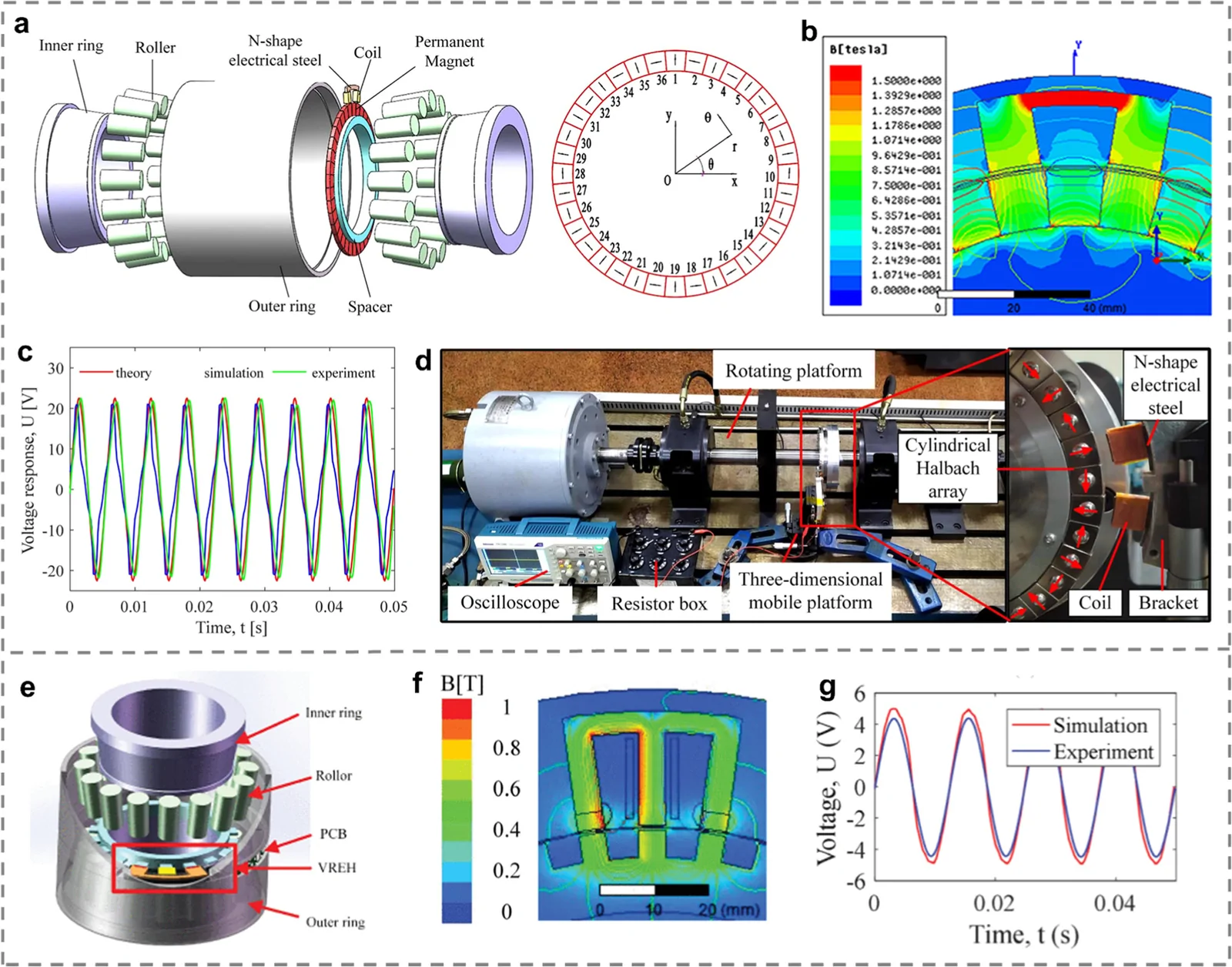
**Tapered roller bearings based on electromagnetic power generation technology.** (a) Schematic diagram of the energy harvester in the smart bearing. (b) Magnetic flux density distribution of the cylindrical Halbach array with n-type electrical steel. (c) Voltage response waveform at a speed of 1200 r/min. (d) Photo of the entire experimental setup [47]. (e) Schematic of the electromagnetic energy-harvesting bearing. (f) Magnetic flux density distribution. (g) Voltage response of the electromagnetic energy harvester at a speed of 1200 r/min [48].

Gong et al. also proposed a variable reluctance energy harvesting system (VREH), which provides self-powering for wireless sensor nodes in intelligent railway axle box bearings. The system consists of the VREH and a power management circuit (PMC) [52]. VREH generates electrical energy through the relative motion within the bearing, while the PMC is responsible for voltage boosting, impedance matching, rectification, and regulation (Fig. 4e). Experimental results show that over a rotational speed range of 400 to 1200 r/min, the power generation and charging efficiency of the VREH system increase with speed. At 1200 r/min, VREH can output 76.05 mW of power, with the PMC achieving a charging efficiency of 52.6%. The working principle of VREH is based on the alignment of toothed pads with E-shaped electrical steel legs, where the magnetic flux is maximized when aligned and obstructed when not aligned, altering the magnetic field distribution (Fig. 4f). At different rotational speeds, the output voltage of VREH follows a sinusoidal waveform, proportional to speed. At 1200 r/min, the error between experimental and simulation results is 14.1%, mainly due to the frequency dependence of inductance (Fig. 4g).

## 3. Piezoelectric power generation technology

Piezoelectric materials can provide high voltage in small ranges, making them particularly suitable for low-frequency and small-amplitude mechanical vibrations. These materials can harness small movements in bearings to generate electrical power. D'Ambrogio et al. [53] successfully prepared a piezoelectric composite material suitable for aerospace bearing monitoring by embedding lead zirconate titanate (PZT) fillers into a polydimethylsiloxane (PDMS) matrix. This composite material was applied to the rings of aircraft ball bearings, resulting in the development of a vibration condition monitoring sensor. The sensor enhances the reliability and safety of bearing operation, continuously monitoring the bearing’s working state in real-time, reducing the occurrence of sudden failures, thus improving efficiency and safety (Fig. 5a). By comparing the response of this vibration sensor with that of a traditional accelerometer-based sensor, it was found that the time series of both signals exhibit similar vibration characteristics, indicating that the piezoelectric material deposited on the bearing ring effectively captures vibration information (Fig. 5b).

**Fig. 5:**
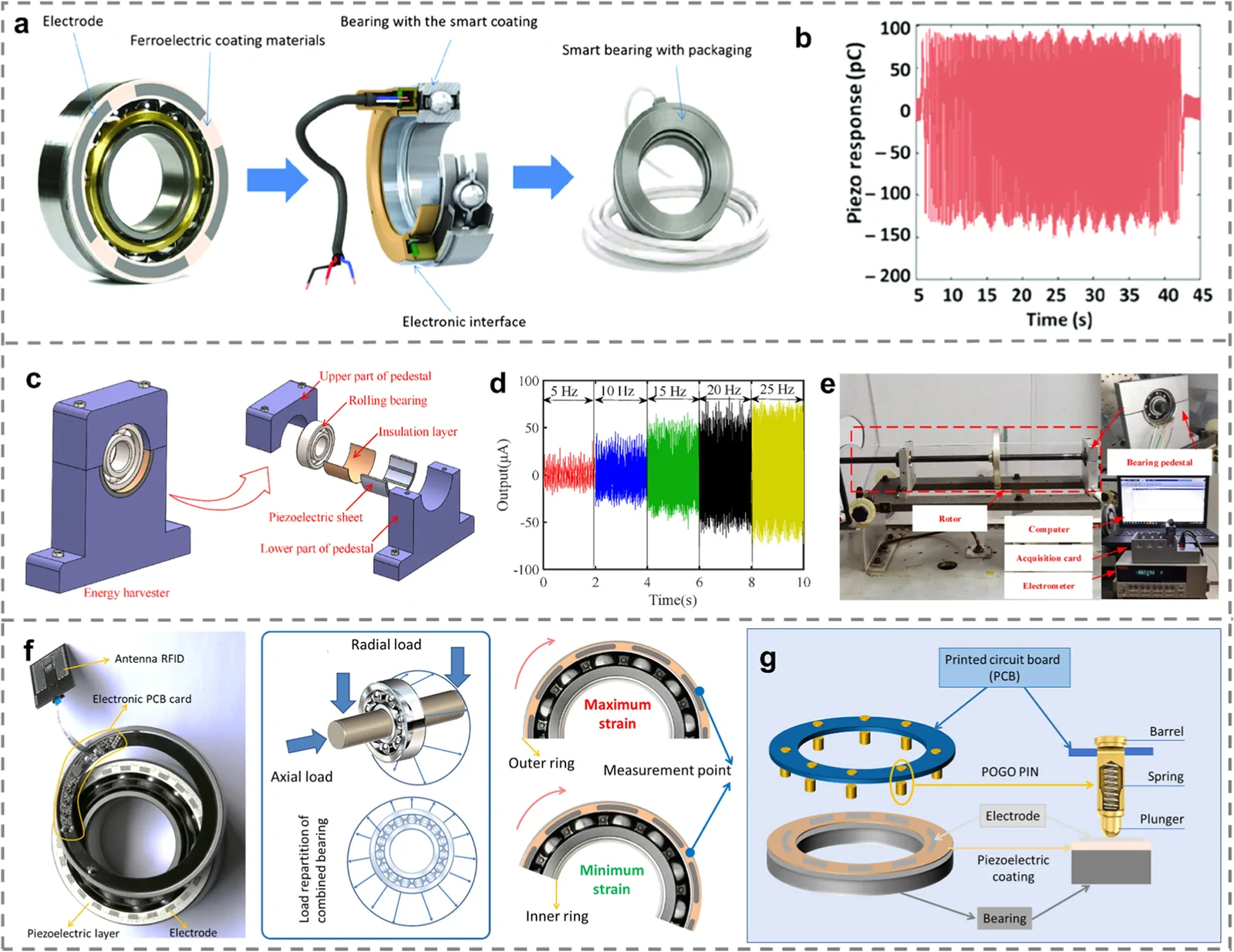
**Piezoelectric power generation technology in self-powered bearings.** (a) Schematic of the piezoelectric coating for bearing condition monitoring. (b) Response curve using the piezoelectric coating mounted on the bearing [49]. (c) Schematic of the piezoelectric energy harvester structure. (d) Short-circuit current of the energy harvester at different rotational frequencies. (e) Photo of the entire experimental setup [50]. (f) Smart piezoelectric sensor integrated into the ball bearing component. (g) Schematic of the wiring for piezoelectric smart sensors with spring loading [51].

Next, Zhang et al. proposed a piezoelectric-based energy harvester installed between the outer race of a rolling bearing and its bearing seat, which collects rotational energy from rotating machinery to power sensors [54]. In the structural schematic of the energy harvester, the arc-shaped piezoelectric plates are mounted on the lower part of the support, with a design ensuring that the installation of the piezoelectric plate does not affect the performance of the rolling bearing. To enhance energy harvesting efficiency, the electrode layers of the piezoelectric plates are cut into multiple conductive parts. The motion of the rolling elements transforms the constant compressive load on the piezoelectric plate into a fluctuating compressive load, further improving the electrical energy conversion efficiency (Fig. 5c). They developed an electromechanical coupling model for the energy harvester and analyzed the effects of various parameters, such as rotational speed, rotor weight, shaft span, and impedance matching, on the harvester's performance. Experimental results showed that, within the rotational speed range of 600 to 1200 r/min, the amplitude of the short-circuit current generated by the harvester increases linearly with speed, indicating that an increase in speed helps to enhance the current output (Fig. 5d). This energy harvester not only powers the sensor but also has the ability to detect bearing faults, verifying its feasibility in practical applications (Fig. 5e).

In the field of intelligent sensor technology, Nguyen et al. proposed a bearing load monitoring technology based on piezoelectric coatings [55]. The piezoelectric ink was screen printed onto the outer bearing substrate. This coating consists of piezoelectric composite layers, dielectric layers, and conductive layers, exhibiting excellent sensing performance. The sensor continuously monitors the changes in bearing load, reflects the bearing's working state in real-time, and triggers an alarm signal when the load exceeds a preset threshold (Fig. 5f). They proposed a novel design method where the electrode size on the bearing’s outer ring is optimized to improve sensor sensitivity while simplifying the printing process. This bearing can accurately detect changes in load, even monitoring the strain of each rolling element (Fig. 5g).

Additionally, Safian et al. proposed an embedded piezoelectric sensor for fault detection in cylindrical roller bearings [56]. By combining a piezoelectric ceramic (PZT) disk with an alumina disk, the sensor can effectively withstand high radial loads without significant stress (Fig. 6a). They developed an equivalent model for this power generation structure, providing theoretical support for the study of piezoelectric sensor performance, and used finite element analysis to study the performance of the generator under various experimental conditions. Experimental results showed that this sensor can successfully detect localized bearing faults via voltage signals (Fig. 6b).

**Fig. 6:**
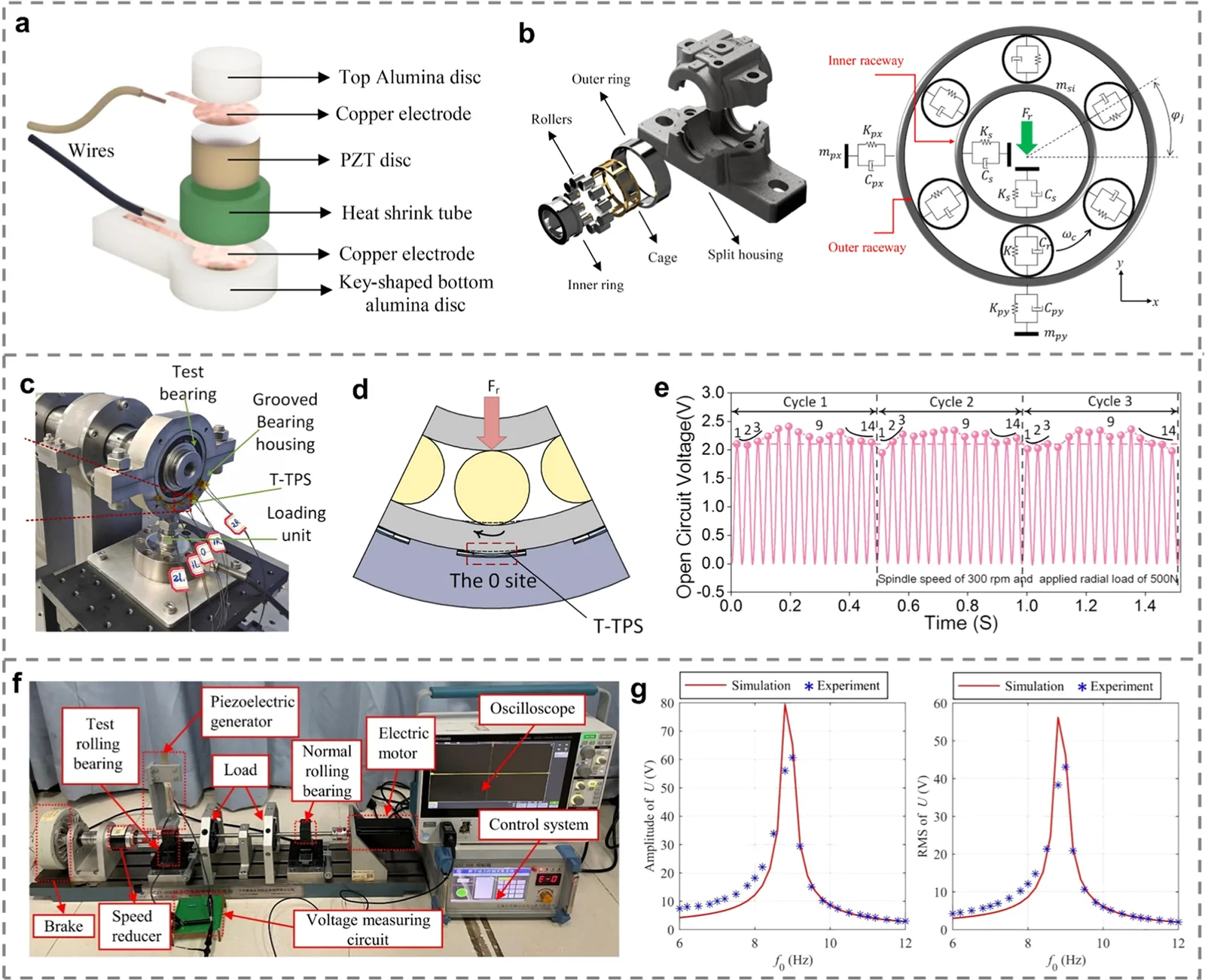
**Self-powered bearings based on piezoelectric power generation technology.** (a) Exploded view of the piezoelectric generator structure. (b) Exploded and equivalent diagram of the roller bearing [52]. (c) Structural diagram of the bearing integrated with T-PPS. (d) Measurement principle for load and speed on the bearing. (e) Voltage signal measured by the piezoelectric generator integrated into the bearing [53]. (f) Experimental setup for rolling bearing fault diagnosis. (g) Output voltage of the piezoelectric generator at different frequencies [54].

Li et al. studied the triboelectric-piezoelectric coupling mechanism at the ferroelectric polymer/metal interface under compression and developed a triboelectric-piezoelectric coupling pressure sensor (T-PPS) using Polyvinylidene fluoride-copper (PVDF-Cu) as an example [57]. The system consists of a T-PPS integrated with the bearing, and a multi-channel signal acquisition circuit. The T-PPS is installed between the bearing’s outer race and the bearing box (Fig. 6c). The operating principle shows that whenever a roller passes, the T-PPS captures pressure fluctuations and converts them into electrical signals (Fig. 6d). The electrical signal graphs under different speeds and load conditions reflect the changes in roller speed and bearing load, providing reference data for sensing. The sensor offers high sensitivity and a wide measurement range, enabling intelligent load distribution and roller speed synchronous online testing in detecting pressure fluctuations on the bearing's outer race (Fig. 6e).

Finally, Shi et al. [58] proposed a self-powered rolling bearing fault diagnosis method based on a piezoelectric generator (PEG). By installing the PEG on the bearing, vibration energy is converted into electrical power to provide electrical signals for fault diagnosis. The PEG is fixed at the 12 o'clock position of the test bearing, and by measuring its electrical output, the vibration characteristics of the bearing can be monitored to diagnose faults (Fig. 6f). The electrical output versus frequency graph of the PEG shows that the simulation curve matches the experimental data, verifying the method's effectiveness. The PEG generates voltage output at different frequencies and can be used for fault diagnosis (Fig. 6g).

## 4. Thermoelectric power generation technology

Thermoelectric power generation technology can directly convert temperature differences into electrical energy with no moving parts in the system, greatly reducing maintenance costs. Zaghari et al. proposed an integrated self-powered wireless system that utilizes a thermoelectric generator (TEG) to supply power, particularly suitable for high-temperature (above 125 °C) environments, used for bearing condition monitoring [59]. Unlike other traditional thermoelectric generators, this system includes an internal oil cooling chamber, allowing for higher energy output under small temperature gradients, such as those in jet engine environments. The system works by placing the TEG on a heat plate, exposing it to different temperature gradient environments, and measuring the thermocouple, along with controlling oil temperature and flow pipeline positions. To simulate the actual working conditions of an engine, the TEG was placed in a chamber, isolated with an oil inlet and outlet pipe (Fig. 7a). By using strain gauges (ZFCAL-1–11 type) and K-type thermocouples, axial load, radial load, and bearing temperature were measured to ensure comprehensive monitoring of the bearing's working state. Experimental results showed that the TEG could provide enough energy for a wireless sensing system in a simulated engine environment, supporting the system to collect data every 46 s during the critical takeoff stage and part of the cruise stage, and transmit data every 260 s. These design solutions demonstrate the broad application potential of thermoelectric power generation technology in self-powered bearing systems (Fig. 7b).

**Fig. 7:**
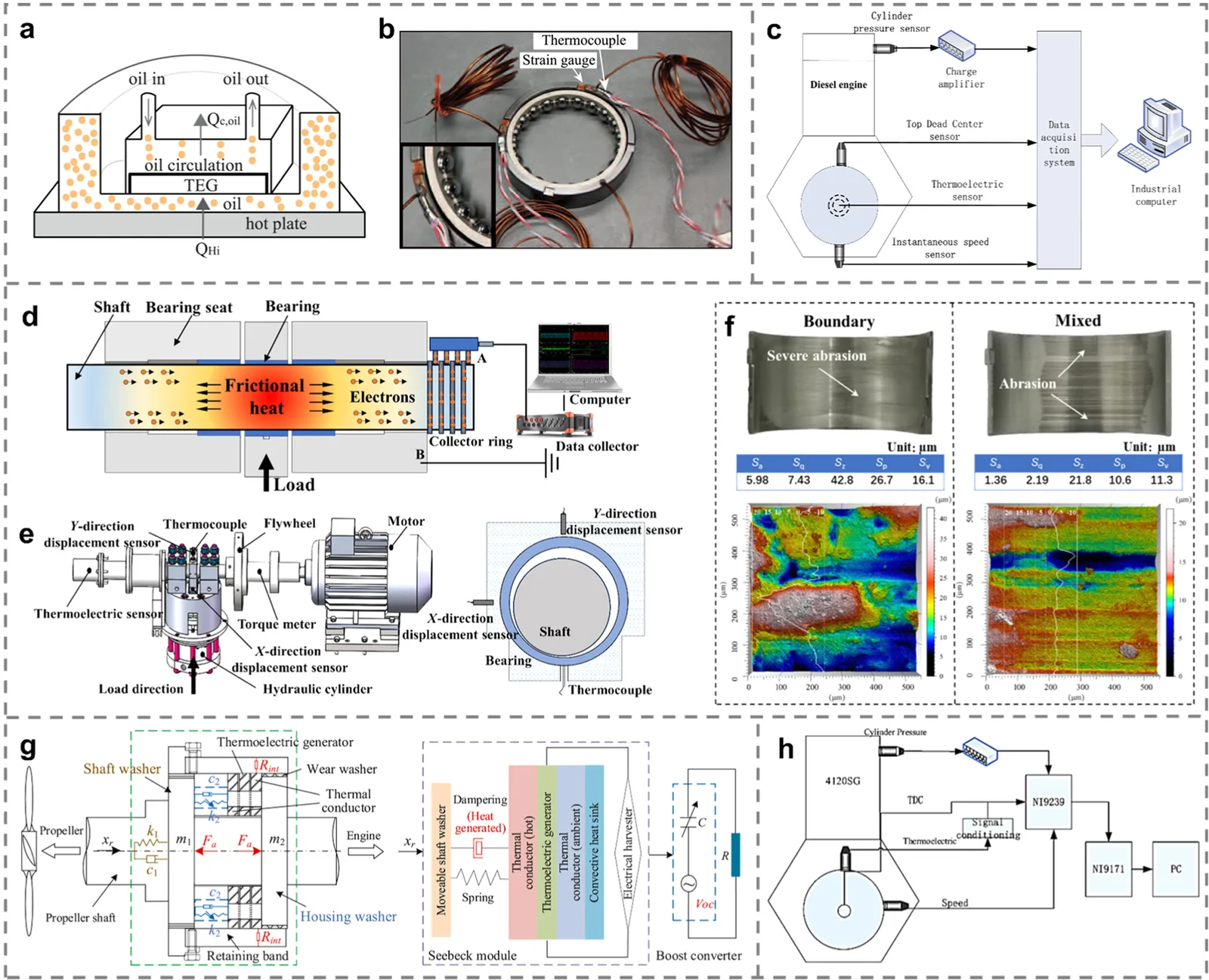
**Self-powered bearings based on thermoelectric power generation technology.** (a) Schematic diagram of the thermoelectric generator with internal cooling oil. (b) Strain gauges and thermocouples mounted on the bearing [55]. (c) Alloy bearing thermoelectric monitoring test system [56]. (d) Schematic diagram of thermoelectric potential measurement. (e) Structure diagram of the bearing test rig. (f) Photo and 3D surface morphology of the bearing wear surface [57]. (g) Schematic diagram of the ship thrust bearing system and vibration converted into electrical energy [58]. (h) Thermoelectric bearing system for diesel engine monitoring [59].

The thermoelectric effect has also been effectively applied in the wear monitoring of alloy bearings. Zhu et al. studied the thermoelectric signal variation characteristics of alloy bearings under different types and working conditions [60]. The results showed that the thermoelectric signals of alloy bearings exhibited significant changes in temperature potential and contact potential. During steady-state operation, these thermoelectric signal variations showed good repeatability. More importantly, the peaks of these thermoelectric signals are associated with the number of working cylinders, while their average values are closely related to the thickness of the oil film. Therefore, by monitoring these thermoelectric signals, it is possible to assess the cumulative and instantaneous wear of alloy bearings in real time, proving the feasibility of using thermoelectric effects for online wear monitoring of alloy bearings (Fig. 7c).

Additionally, Lv et al. proposed a novel thermoelectric monitoring method for determining the lubrication state of bearings [61]. The study revealed a strong correlation between the thermoelectric potential of bearings and their lubrication state, with temperature and film thickness being the key factors influencing the thermoelectric potential (Fig. 7d). Through the study of thermoelectric potentials under different lubrication conditions, the impact of lubrication state on bearing performance was further confirmed. This result was validated using a Stribeck curve. In further experiments, they simulated different lubrication conditions using a bearing test rig, hydraulic loading system, and motor speed control device (Fig. 7e). They also used a confocal laser scanning microscope (CLSM) to capture images and conduct three-dimensional surface analysis of the bearing surface, revealing the surface changes under different lubrication conditions. This series of research results provides valuable experimental data for the application of thermoelectric technology in bearing lubrication state monitoring (Fig. 7f).

In the field of thermoelectric energy harvesting for thrust bearings, Huang et al. designed a TEG that converts vibration heat into electrical energy for energy collection in thrust bearings. The system demonstrated excellent performance when compared with existing technologies [62]. The study also analyzed the dynamic responses of spring force and damper force in the bearing races at different rotational speeds, explored the relationship between temperature variation and thermal power in the thermoelectric conversion process, and evaluated the output power of the thermoelectric generation system. By adjusting system parameters, efficient conversion and collection of vibration energy can be achieved. This technology not only improves bearing energy efficiency but also provides continuous energy support for thrust bearing condition monitoring (Fig. 7g).

Furthermore, Biao et al. [63] proposed new research on the application of thermoelectric methods in monitoring wear in diesel engine main bearings. Through variable load tests on a sapphire sliding bearing test rig, they investigated the relationship between thermoelectric signals and bearing wear. Experimental results showed that as wear increased, the amplitude of the thermoelectric signals gradually increased, demonstrating the close correlation between thermoelectric signals and bearing wear. To further improve diagnostic accuracy, the researchers used the TrAdaBoost learning algorithm to successfully build a diagnostic model capable of identifying wear conditions under different load scenarios of diesel engine main bearings, achieving an accuracy rate of over 95%. This research suggests that thermoelectric signals can become an important tool for monitoring bearing wear, offering new ideas for bearing health monitoring and maintenance (Fig. 7h).

## 5. Triboelectric power generation technology

A triboelectric nanogenerator generates electric charge through friction or contact separation between two different materials. Common materials used include polymers, metals, textiles, etc., which are not only widely available but also low-cost to manufacture. Choi et al. proposed a triboelectric sensor based on a rolling bearing structure, employing a combination of rollers and electrodes to generate electrical signals when the bearing rotates [64]. This design can provide an open-circuit voltage of about 45 V and a short-circuit current of around 6 µA at a speed of 400 rpm, indicating its potential for self-powering low-power devices. With this triboelectric sensor, smart bearings can monitor the angular velocity, position, and damage status of components in real time, extending the application of triboelectric nanogenerators in self-powered sensing systems (Fig. 8a).

**Fig. 8:**
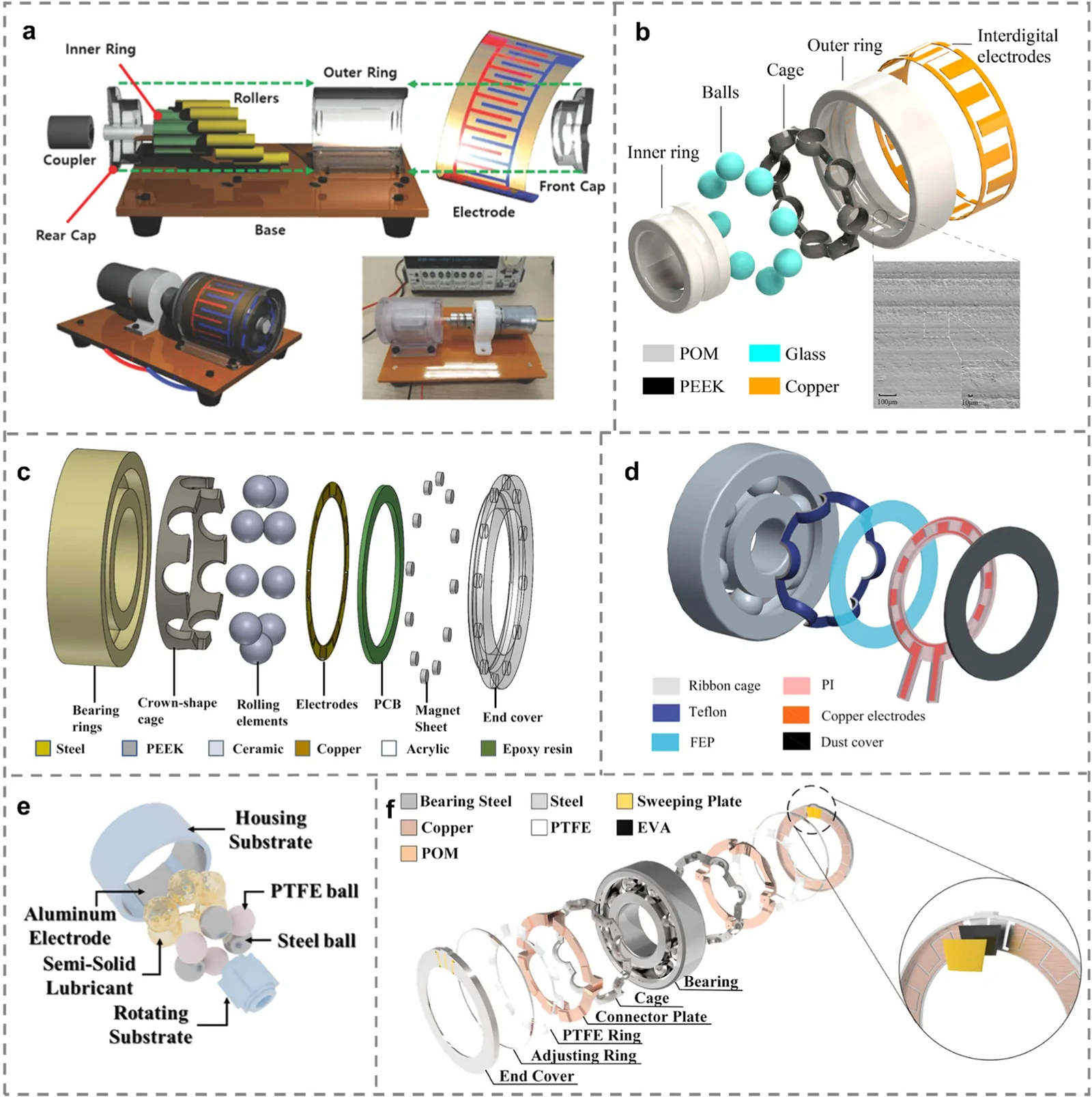
**Triboelectric power generation technology in self-powered bearings.** (a) Multifunctional self-powered smart bearing based on triboelectric generator [60]. (b) Triboelectric rolling ball bearing based on rolling independent mode triboelectric nanogenerator [61]. (c) High-speed hybrid ceramic rolling-element friction bearing based on crown-shaped cage and floating rotating-sliding independent mode [62]. (d) Friction bearing based on strip-shaped cage [63]. (e) Rolling ball bearing-type friction nanogenerator with semi-solid lubricant [64]. (f) Non-contact friction bearing sensor [65].

The triboelectric rolling ball bearing (TRBB) proposed by Han et al. showcases another innovative self-powered technology [65]. This technology avoids direct contact between the electrode and the rolling ball by attaching flexible electrodes to the bearing’s outer ring, thus ensuring the bearing's longevity. TRBB exhibited excellent electrical output characteristics at various speeds, generating stable electrical signals and optimizing design based on different rotational speeds and load resistances. Furthermore, TRBB has the ability for self-sensing of abnormal states, such as the loss of rolling balls or electrode damage, and can self-detect and output relevant signals. This multifunctional self-powered bearing has wide applications in the Internet of Things (IoT) (Fig. 8b).

Gao et al. developed a high-speed hybrid ceramic rolling element triboelectric bearing (US-HCTEB) for real-time dynamic behavior and stability monitoring [66]. The bearing adopts a floating rolling-sliding combined independent mode to ensure structural integrity and achieves triboelectric output by coupling with sector-shaped electrodes. US-HCTEB can operate at ultra-high speeds of up to 16,000 rpm and maintain high reliability even after 30 million cycles. This technology not only enables monitoring of dynamic behavior before bearing failure but also provides high-resolution real-time data, which is crucial for the development of smart rolling bearings (Fig. 8c).

Jiang et al. proposed a triboelectric bearing with a belt-shaped retainer (RTB) for fault diagnosis of rotating machinery [67]. By spraying a Teflon insulating coating on the surface of the belt-shaped retainer and attaching electrodes inside the dust shield, a freestanding triboelectric self-sensing system was formed. RTB can monitor faults such as localized damage to gears or bearings by measuring the variation in output current. After combining with time-frequency transformations and deep learning algorithms, RTB can achieve high-precision fault diagnosis, showing significant potential for industrial applications (Fig. 8d).

Song et al. demonstrated a rolling ball bearing-type triboelectric nanogenerator combined with commercial semi-solid lubricants. By using non-polar semi-solid lubricants, the electrical output performance and mechanical life of the bearing were significantly improved [68]. This design reduces wear and enhances the triboelectric effect, producing stable electrical output over 55 h of continuous operation. Additionally, the design uses PTFE and stainless steel balls to prevent thermal damage, ensuring high electrical output at high speeds (Fig. 8e).

Xie et al. [69] proposed a non-contact triboelectric bearing sensor (NC-TEBS) that employs a non-contact sensing electrode structure for monitoring a wide range of speeds. NC-TEBS can monitor bearing speed and slip conditions in real time across a range from low speeds to 5000 rpm, with high precision and stability. After 5 million continuous rotations, NC-TEBS still maintains high output performance, indicating its excellent lifespan and reliability. This sensor provides both theoretical and experimental foundations for the development of new self-powered and self-sensing smart bearings (Fig. 8f).

Hu et al. designed a self-powered bearing system based on a triboelectric nanogenerator, using a standard 6204 deep groove ball bearing and two flexible fingertip electrodes mounted on the outer ring [70]. The friction between the rolling elements inside the bearing and the electrodes generates charge, forming an AC signal through the potential difference between the electrodes, enabling self-sensing capability. In this system design, variations in triboelectric signals are used to detect rotor system faults and are analyzed through a deep learning model, achieving a diagnostic accuracy of 90.28%. This design not only accurately identifies misalignment faults in different rotor systems but also improves diagnostic accuracy to 95.76% through ensemble learning. Compared to traditional sensor installation and complex transmission paths, the triboelectric nanogenerator technology offers a simpler and more effective monitoring solution, demonstrating its potential in rotor system applications, especially for bearing condition monitoring without additional sensors (Fig. 9a).

**Fig. 9:**
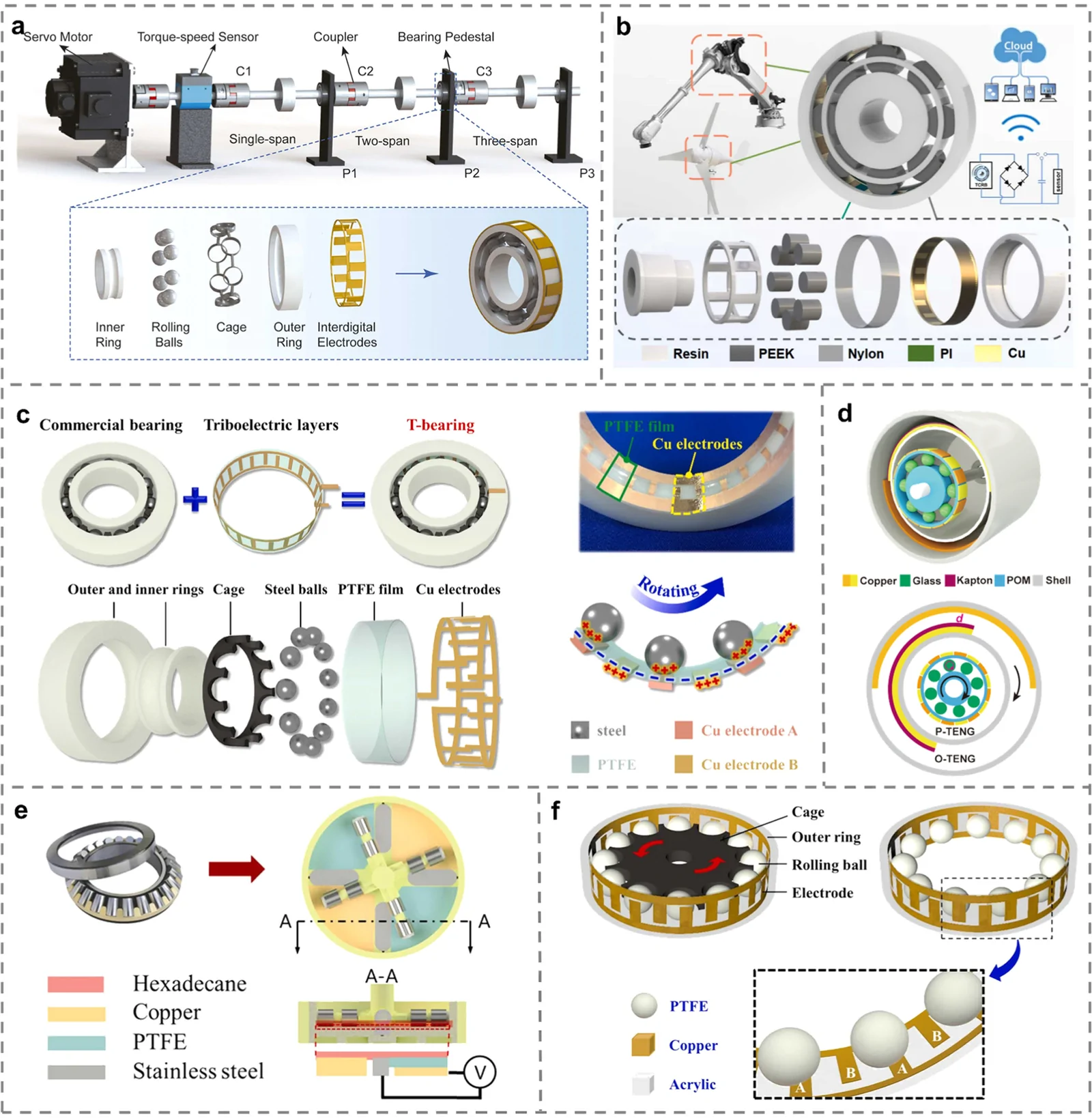
**Self-powered bearings based on triboelectric power generation technology.** (a) Standalone triboelectric nanogenerator bearing [66]. (b) Embedded smart friction nanogenerator cylindrical roller bearing [67]. (c) Embedded triboelectric sensor rolling bearing [68]. (d) High-performance rotating triboelectric nanogenerator for bearing charge pumping [69]. (e) High-sensitivity self-inductive smart thrust roller bearing based on triboelectric nanogenerator [70]. (f) Rolling bearing based on triboelectric nanogenerator [71].

In contrast, Xin et al. proposed an embedded cylindrical roller bearing (TCRB) design using PEEK (polyetheretherketone) material for the cylindrical rollers and the outer ring covered with a nylon-coated grid [71]. TCRB operates stably at an open-circuit voltage of 26.56 V and a short-circuit current of 2.45 µA, generating sufficient power at 600 rpm to drive small sensors. This design also features self-powered monitoring, enabling real-time speed monitoring with an error rate of within 2%. Moreover, TCRB can accurately diagnose bearing faults using triboelectric signals, demonstrating excellent performance in self-powering, self-sensing, and self-diagnosis (Fig. 9b).

Dong et al. proposed a triboelectric sensor embedded in rolling bearings for monitoring the working state of rolling balls and conducting defect diagnosis [72]. This design used copper electrodes with PTFE (polytetrafluoroethylene) films and connected them directly to the inner surface of the bearing's outer ring. Friction between the rolling balls and electrodes generates charge and outputs electrical signals through the electrodes. To enhance detection accuracy, this design combined signal decomposition techniques with machine learning algorithms, achieving high-precision diagnosis of rolling balls under five different operating conditions with an identification accuracy of 99.48%. This design not only increases the sensitivity of triboelectric sensors but also successfully overcomes the problem of excessive signal noise in traditional methods (Fig. 9c).

Furthermore, Fu et al. [73] proposed a robust, high-performance rotating triboelectric nanogenerator (R-TENG) based on bearing charge pumping. The design includes a pump triboelectric nanogenerator (P-TENG), an output triboelectric nanogenerator (O-TENG), a voltage multiplier circuit (VMC), and a buffer capacitor. The output charge from the P-TENG is pumped to the O-TENG, enhancing power output. The R-TENG increased instantaneous power by 32 times below 300 rpm and maintained 95% efficiency after 6.4 × 10⁶ cycles. This design effectively reduces mechanical wear, improves durability, and provides a reliable solution for triboelectric power generation in high-load environments (Fig. 9d).

Wang et al. developed a smart thrust roller bearing (SS-TRB) based on a triboelectric nanogenerator for real-time monitoring of lubricant status [74]. SS-TRB uses four thrust cylindrical rollers, a cage, and a PTFE insulating film to sense contaminants in the lubricant through triboelectric signals. This design exhibits high sensitivity for detecting contaminants such as stainless steel powder, carbon powder, alcohol, and moisture in the lubricant, especially responding precisely to changes in moisture content. Even the smallest intrusion of 0.01% moisture triggers a significant voltage change. This capability makes SS-TRB highly promising for use in harsh applications, particularly in heavy underwater equipment (Fig. 9e).

Finally, Dong et al. proposed an artificial intelligence-driven rolling triboelectric nanogenerator (B-TENG) for bearing wear condition monitoring and fault diagnosis [75]. The design includes the outer ring, rolling balls, and cage, with two sets of forked copper electrodes, forming a freestanding triboelectric nanogenerator with 12 PTFE balls. B-TENG uses rolling balls as direct sensors, combined with signal decomposition methods and deep learning algorithms to monitor and classify different wear types and their severity in real-time. The study shows that the classification accuracy of wear types using B-TENG ranges from 95.20% to 98.40%, significantly outperforming traditional methods. The successful application of this technology enables more accurate detection of bearing wear, fault evolution, and health status, providing a new solution for early fault diagnosis and lifespan prediction of bearings (Fig. 9f).

## 6. Tribovoltaic power generation technology

A Tribovoltaic Nanogenerator (TVNG) is an extension of the triboelectric nanogenerator. The triboelectric nanogenerator is limited by insulating materials and the finite surface charge density, making it difficult to overcome its typical characteristics of low current density and high impedance. Additionally, incorporating periodic metal/polymer structures into bearings based on triboelectric nanogenerators increases system complexity, and soft polymer materials are prone to damage. In 2020, Zhang et al. [45] introduced a new electromechanical energy conversion mechanism based on triboelectric generation using silicon, experimentally validating the tribovoltaic effect. Compared to the triboelectric generation phenomenon in insulating materials, the current density increased by 3–6 orders of magnitude, the impedance decreased by 3–6 orders of magnitude, and direct current was generated, achieving a fundamental breakthrough in power generation performance and a significant increase in efficiency.

Our team proposed a polycrystalline silicon-based TVNG, which demonstrates its potential for in-situ rotational speed monitoring in bearings [76]. While traditional monocrystalline silicon TVNGs have excellent electrical performance, their high cost and brittleness limit their widespread application. In contrast, polycrystalline silicon TVNGs are not only more cost-effective but also offer electrical output performance similar to monocrystalline silicon. The grain boundaries in polycrystalline silicon can suppress carrier transport but also provide additional carriers, allowing stable electrical output when the rotational speed changes. Results show that polycrystalline silicon-based conical roller bearings, thrust ball bearings, and deep groove ball bearings can achieve good rotational speed monitoring. For example, the open-circuit voltage (V_oc_) and short-circuit current (I_sc_) of the conical roller bearing increase linearly from 0.19 mV and 0.035 µA to 0.09 mV and 0.08 µA in the speed range of 70 rpm to 250 rpm (Fig. 10a). Similarly, the electrical output of thrust ball bearings and deep groove ball bearings maintains a linear relationship with rotational speed (Fig. 10, Fig. 10). Based on this linear relationship, our team developed an in-situ speed self-sensing bearing system. After wireless transmission and data processing of the electrical signals from the self-powered bearings, real-time monitoring of the bearing's rotational speed is achieved, providing early warnings in case of abnormalities. This in-situ speed self-sensing smart bearing system demonstrates the huge potential of TVNG in industrial IoT applications (Fig. 10d).

**Fig. 10:**
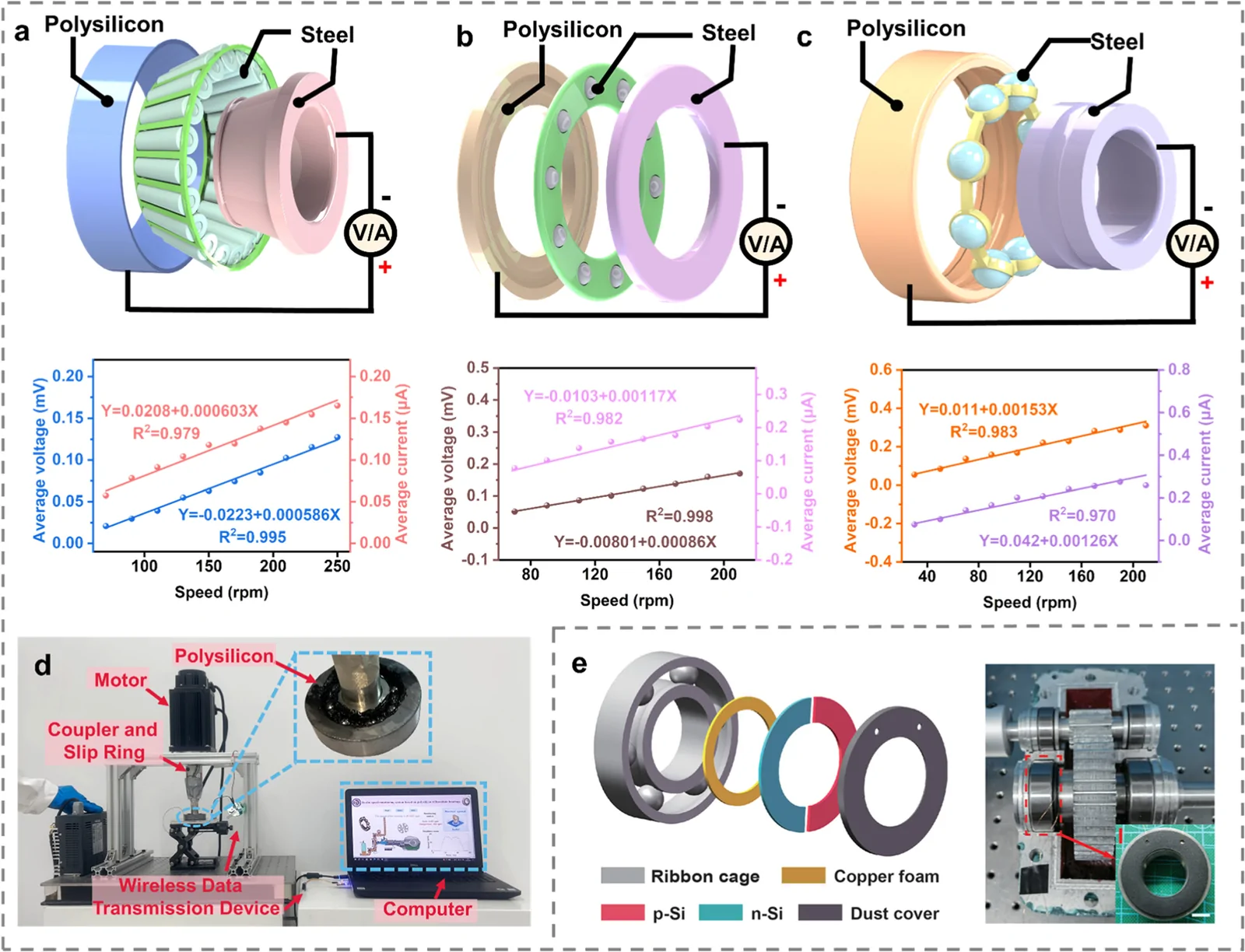
**Tribovoltaic power generation technology in self-powered bearings.** (a) Cone roller bearing based on polycrystalline silicon tribovoltaic nanogenerator (Si-TVNG) and its electrical output. (b). Thrust ball bearing based on Si-TVNG and its electrical output. (c) Deep groove ball bearing based on Si-TVNG and its electrical output. (d) Self-powered rotational speed sensing bearing system [72]. (e) Freestanding silicon-based tribovoltaic bearing [73].

In addition, Dong et al. proposed a freestanding TVNG (FTVNG) based on metal, p-type silicon (p-Si), and n-type silicon (n-Si), which facilitates energy collection within the bearing system [77]. The uniqueness of FTVNG lies in the fact that it simultaneously contacts both p-Si and n-Si through friction, generating a forward DC current (from p-Si to n-Si) in the external circuit. This design not only effectively collects mechanical energy but also solves the complex problem of lead rotation with the shaft in traditional TVNG power generation systems, as the friction effect between metal and semiconductor parts generates current in a freestanding mode. FTVNG can be integrated into traditional rolling bearings and is highly adaptable in industrial environments. By precisely controlling the contact area with steel pins inside the bearing, researchers further explored the effects of working parameters such as friction area and rotational speed on electrical output. Results show that FTVNG can serve not only as an energy collector for bearings but also for real-time speed monitoring, making it suitable for high-speed rotating bearing systems. This innovation provides important theoretical and practical support for self-powered sensing systems in bearings, especially in the smart monitoring of industrial equipment, with a wide range of application prospects (Fig. 10e).

Based on the freestanding structure mentioned above, Yu et al. proposed a TVNG-based double-row tapered roller smart bearing (TVB) [78]. TVB generates electrical energy during friction by combining two differently doped monocrystalline silicon rings as stators with bearing rollers to monitor rotational speed. Its most notable feature is the adoption of a freestanding mode structure, which allows electrodes to be drawn from the stator side, thus solving the problem of wiring for the moving part of the bearing. This design enables the bearing to convert friction into electrical energy without additional wiring while monitoring speed in real-time (Fig. 11a). To further optimize the system, Yu et al. encapsulated the signal processing unit along with the TVB in an integrated module, forming an electromechanical smart bearing system (Fig. 11b). The results showed that in the speed range of 400 rpm to 1000 rpm, the current of TVB had a good linear relationship with the speed (R^2^ values of 0.9933 and 0.9867), allowing precise monitoring of the bearing's speed and over-speed alarm. This system enables real-time speed monitoring and provides an alert mechanism to remind operators to perform maintenance or replacement in a timely manner. This innovative design provides a more smart monitoring approach for industrial equipment, especially in industrial environments requiring high reliability and real-time response, and has significant application value (Fig. 11c).

**Fig. 11:**
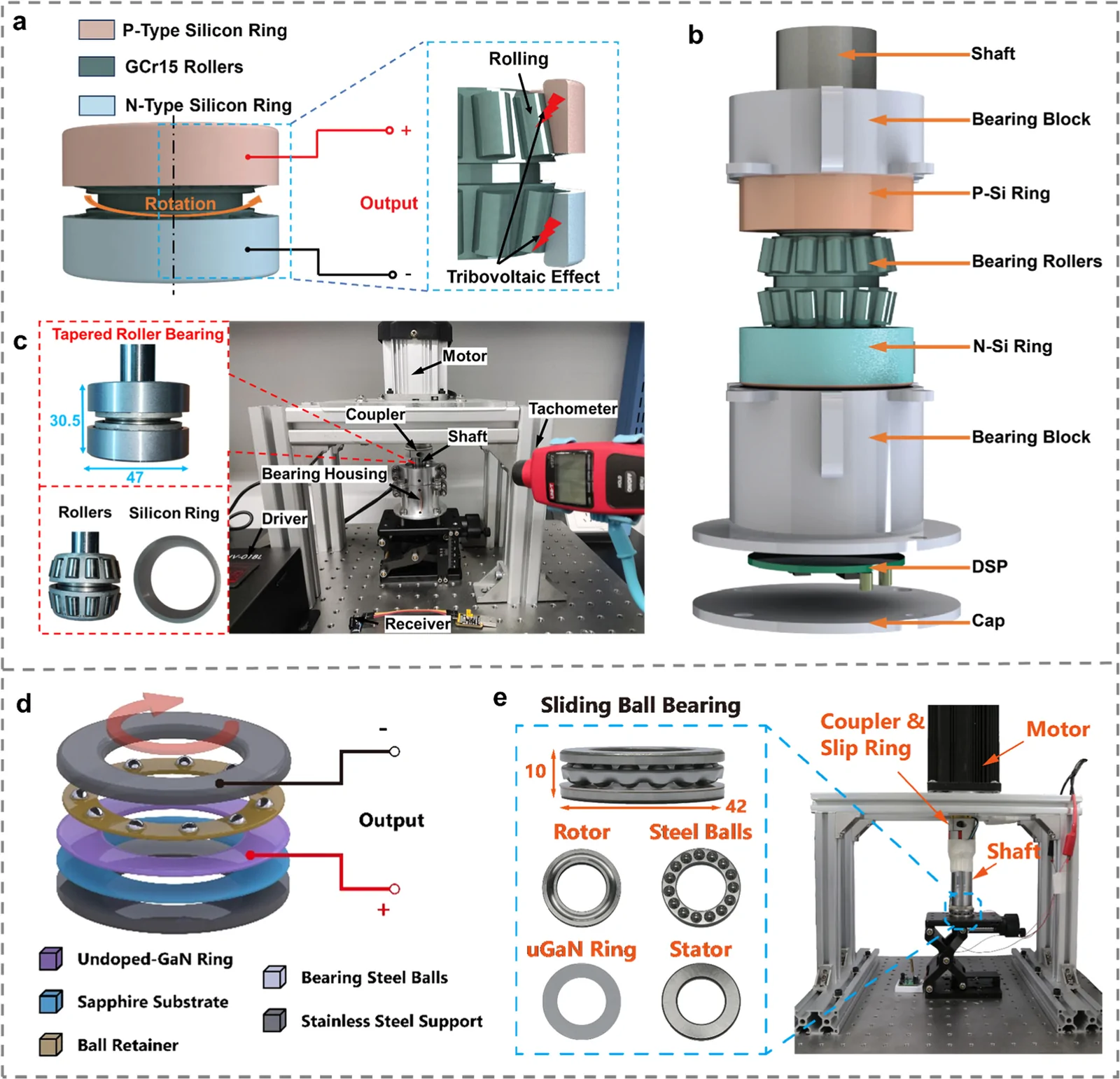
**Self-powered bearings based on tribovoltaic power generation technology.** (a) Structure of the standalone tribovoltaic bearing. (b) Integrated structural-functional standalone tribovoltaic bearing system. (c) Rotational speed sensing system based on standalone tribovoltaic bearing [74]. (d) Schematic diagram of thrust ball bearing based on tribovoltaic. (e) Self-driven tribovoltaic thrust ball bearing system [21].

In terms of high-performance energy generation, Zhang et al. created a TVNG with ultra-high standardized average power density and low wear rate by using a gallium nitride (GaN) and steel ball-based ball-disk structure [21]. The study showed that this GaN-based TVNG achieved an open-circuit voltage of over 130 V and a power density of 24.6 kW/m²·Hz during friction, improving by 282 times compared to previous works. Moreover, the GaN-based TVNG also exhibited a low wear rate, maintaining stable electrical output performance even under maximum contact pressure. This research not only demonstrates the advantages of GaN material in TVNGs but also provides technical support for self-powered sensor nodes in industrial IoT. On this basis, the research team designed the first GaN-based triboelectric sliding ball bearing and successfully applied it to a real-time environmental monitoring system (Fig. 11d). By integrating an energy management module, data collection module, and transmission module, this system can transmit sensor signals at a speed of 300 rpm within 16 s. This technological breakthrough enables the bearing system to monitor important environmental parameters such as pressure and temperature in real time and transmit data for processing and feedback wirelessly. This system not only enhances the efficiency of triboelectric energy recovery but also expands its application potential in smart manufacturing and industrial automation (Fig. 11e).

## 7. Comparison and discussion of self-powered technologies in bearings

Electromagnetic, piezoelectric, thermoelectric, triboelectric, and tribovoltaic power generation technologies each have unique features in their applications within bearing systems. Their advantages and disadvantages determine their ability to adapt to different working conditions and requirements (Table 1).

**Table 1:** **Comparison of five self-powered bearing technologies**.

	Electromagnetism	Piezoelectricity	Thermoelectricity	Triboelectric	Tribovoltaic
Principle	Electromagnetic induction law	Piezoelectric effect	Thermoelectric effect	Contact electrification and electrostatic coupling	Tribovoltaic effect
Discovery time	1831	1880	1821	2012	2020
DC or AC	AC	AC	DC	AC	DC
Voltage	1–10V	1V-100V	1–10V	10V-1000V	0V-150V
Impedance	10–100 Ω	1 kΩ−100 kΩ	10 Ω−100 Ω	1MΩ−1GΩ	10Ω−10MΩ
Advantage	Reliable and stable	Collecting small vibrations with a compact structure	No moving parts, low maintenance cost	Wide range of materials, low cost	Simple structure and strong wear resistance
Disadvantage	Complex structure, not suitable for miniaturization	High impedance, requiring circuit management	Limited temperature difference, low output power	High impedance, material prone to wear and tear	Low energy conversion efficiency

Electromagnetic power generation technology is based on Faraday’s law of electromagnetic induction and is suitable for high-speed bearing systems. Its working principle relies on Faraday’s law of electromagnetic induction, generating electricity through relative motion. It typically outputs AC, with a voltage range between 1 and 10V and an impedance range of 10–100Ω [[79], [80], [81]]. The advantages of electromagnetic power generation lie in its ability to provide stable voltage and current outputs, making it suitable for bearing monitoring systems that require a reliable power source. This technology is applicable to high-speed rotating bearing systems, efficiently recovering mechanical energy. However, the disadvantage of electromagnetic power generation is its complex structure, requiring intricate electromagnetic coils and magnet configurations, with efficiency constrained by rotational speed and magnetic field strength. Thus, it is not suitable for space-constrained or miniaturized applications. At the same time, the counter-torque in the bearings of electromagnetic generators increases the load on the bearings, resulting in additional energy losses.

Piezoelectric power generation technology converts small vibrations or movements in bearings into electrical energy through the piezoelectric effect, particularly suitable for low-frequency vibrations and small-amplitude movements. Its typical output voltage range is 1V-100V, with an output of AC and high impedance, usually between 1kΩ−100kΩ [82,83] The advantage of piezoelectric power generation is its ability to collect energy from small movements in bearings, making it ideal for long-term monitoring and sensing applications. Its compact structure makes it well-suited for environments with limited space, especially in bearing health monitoring systems. However, its disadvantage lies in the system’s dependence on high impedance, which may require energy storage batteries or circuitry to improve stability. Additionally, piezoelectric materials exhibit the highest energy conversion efficiency near their resonant frequency, which typically lies in the kHz range or above. However, since bearing operation frequencies generally range from tens to hundreds of Hertz, structural design optimizations are required to bridge this frequency gap.

Thermoelectric power generation technology utilizes the temperature difference generated during the bearing operation and converts it into electrical energy through the thermoelectric effect, outputting DC. The typical voltage range is 1V-10V, with impedance typically between 10Ω−100Ω [34,35]. The advantage of thermoelectric power generation is that it has no moving parts, ensuring stable long-term operation, making it highly suitable for applications in environments with temperature differences. However, the primary drawback of thermoelectric technology is its reliance on the temperature difference, and the small temperature differences often lead to low power output, especially in situations where bearing temperature variations are minimal, resulting in low energy collection efficiency.

Triboelectric power generation technology generates electrical charges through friction and contact on the bearing surface and collects energy via electrostatic coupling principles, typically outputting AC. The typical voltage range is 10V-1000V, with high impedance, typically between 1MΩ−1GΩ [84,85]. The advantages of triboelectric power generation include a wide range of material choices, simple structure, and low manufacturing costs, making it suitable for bearing health monitoring and low-cost applications. However, its disadvantages include a high dependence on materials and surface conditions, and the wear of the friction pair may impact long-term stability [86]. The high impedance requirements may necessitate circuitry adjustments to balance voltage and current output, adding complexity to the system. Moreover, it is necessary to design periodic structures within the bearings, which increases the complexity of the bearing structure.

Tribovoltaic power generation technology utilizes the tribovoltaic effect by exciting carriers through the friction of semiconductor materials to output DC. The typical voltage range is 0V-150V, with impedance between 10Ω−10MΩ [[87], [88], [89]]. The advantages of tribovoltaic power generation include its ability to directly generate energy at the interface of the friction pair, achieving self-powering and monitoring systems. Due to its simple structure and direct energy harvesting at the friction interface, it offers strong compressive strength and durability, making it suitable for in-situ sensor monitoring [90,91]. However, the energy conversion efficiency of tribovoltaic power generation is relatively low, requiring continuous optimization of friction material selection and interface design to improve energy conversion efficiency. Additionally, the tribovoltaic effect requires high hardness for the friction materials, and semiconductor materials tend to be more expensive.

In summary, each of these five technologies has its strengths and weaknesses. Electromagnetic power generation is suitable for high-speed and stable bearing monitoring systems, while piezoelectric power generation is ideal for energy harvesting from low-frequency vibrations and small movements. Thermoelectric power generation is well-suited for applications involving significant temperature differences, while triboelectric and tribovoltaic power generation are more flexible and suitable for miniaturized applications. In self-powered bearing systems, the selection of the appropriate technology must be based on the specific requirements of the application environment, balancing factors such as system efficiency and stability.

## 8. Conclusion and outlook

With the continuous advancement of self-powered technologies, their potential applications in smart bearings are gradually being realized. Although several energy harvesting technologies, such as electromagnetic, piezoelectric, thermoelectric, triboelectric, and tribovoltaic generation, have successfully extracted energy from rotational motion, their practical applications in self-powered smart bearings still face some limitations. These limitations continue to hinder the widespread use and performance improvement of self-powered smart bearings. Therefore, overcoming these bottlenecks will be key to future research and technological development (Fig. 12).

**Fig. 12:**
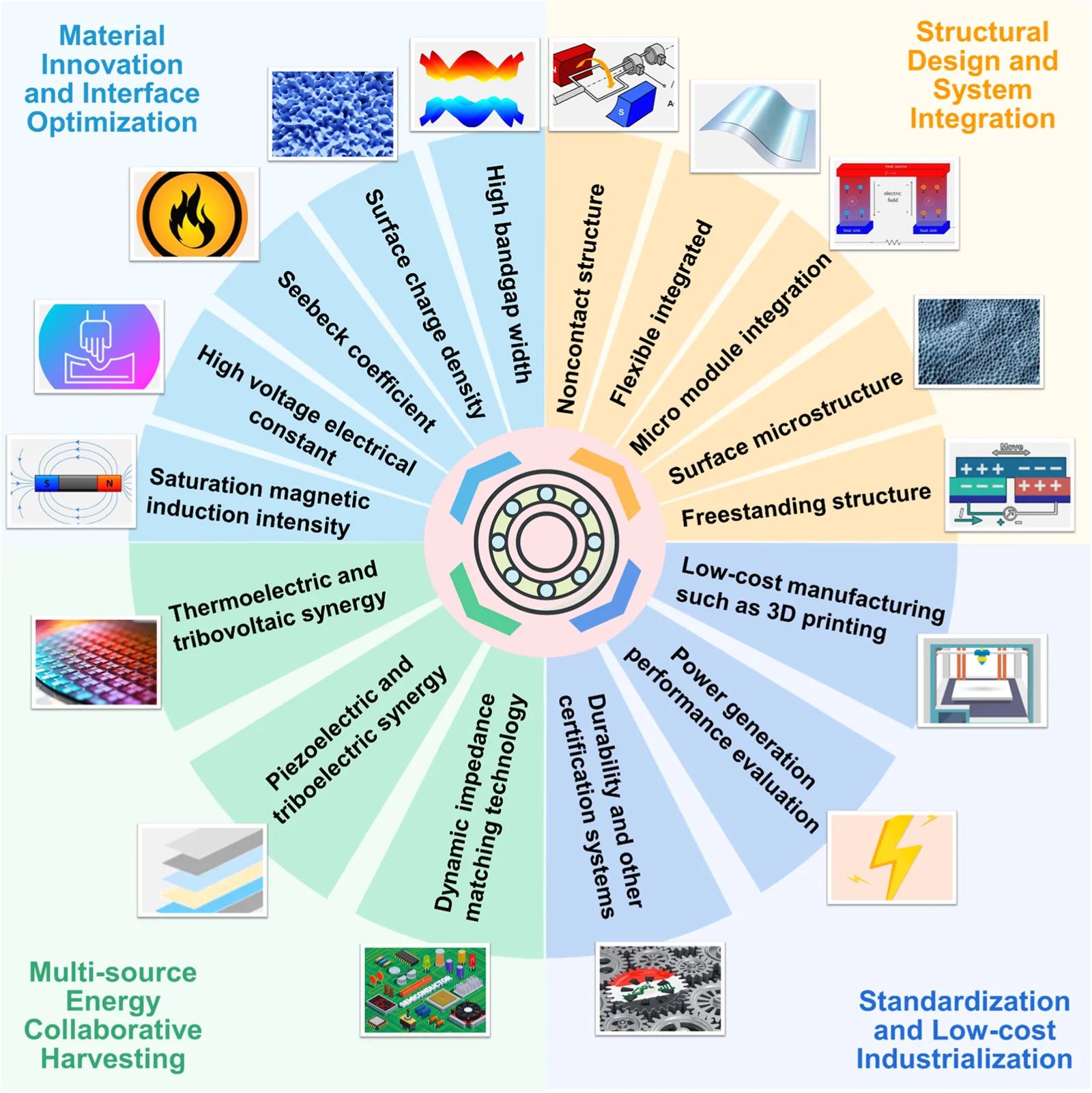
**Outlook on the self-powered technology of smart bearings**.

### 8.1. Material innovation and interface optimization

In future research, material innovation and interface optimization will be key factors in improving the performance of self-powered bearing systems. Different energy harvesting technologies have different material requirements, so materials need to be specifically optimized in each of the five self-powered technologies to enhance energy conversion efficiency, durability, and stability.

For electromagnetic generation technology, future research should focus on improving the saturation magnetic induction intensity and conductivity of magnetic materials to enhance energy conversion efficiency. Specifically, for miniaturized electromagnetic generators, using high-performance soft magnetic materials and low-loss conductive materials (such as high-conductivity copper or aluminum alloys) can significantly improve output power. In the future, innovative magnetic nanocomposite materials and self-healing coating materials may become breakthroughs for enhancing the stability and lifespan of electromagnetic generators.

For piezoelectric generation technology, future research needs to focus on developing piezoelectric materials with high piezoelectric constants and good mechanical properties, such as efficient, flexible piezoelectric polymers and new inorganic piezoelectric materials with a perovskite structure. Additionally, interface optimization between piezoelectric materials and substrates should not be overlooked. Improving adhesion and elastic matching between the material and substrate can effectively enhance the piezoelectric effect.

For thermoelectric generation technology, the core issues are the thermoelectric efficiency of the material and the stability of high temperature differences. In the future, optimizing the electrical conductivity, thermal conductivity, and Seebeck coefficient of thermoelectric materials using nanostructures and doping techniques will be key to improving thermoelectric performance. Furthermore, interface optimization is also important, especially by using heavy doping methods to treat the interface between thermoelectric films and metal layers to reduce thermal resistance and improve overall thermoelectric conversion efficiency.

In triboelectric generation technology, material innovation is especially crucial. Triboelectric performance is closely related to surface charge density, the material's friction coefficient, and surface morphology. Future research could design materials with high surface charge accumulation capacity, such as polymers with high dielectric constants or carbon-based materials (e.g., graphene, carbon nanotubes), to improve triboelectric performance. Additionally, the wear resistance of materials is an important factor affecting the long-term stability of triboelectric nanogenerators, so developing friction materials with high wear resistance or non-contact triboelectric generation technology is also a key area of future research.

For tribovoltaic generation technology, interface optimization and the selection of semiconductor materials will be critical to future research. Techniques such as gradient doping, surface passivation, and crystal structure regulation can be employed to enhance carrier excitation efficiency, thus improving power density. For example, GaN, a wide-bandgap semiconductor material, has demonstrated excellent performance in tribovoltaic generation, and future research could explore other wide-bandgap semiconductor materials (e.g., silicon nitride, aluminum nitride) to further break through the existing power density limits.

### 8.2. Structural design and system integration

In the development of self-powered smart bearings, structural design and system integration are key factors for improving performance and efficiency. Achieving efficient energy harvesting while ensuring the normal operation and mechanical performance of the bearing is one of the core research areas. Different energy harvesting technologies have different structural designs and integration requirements, so future designs will need to focus on optimizing the integration of each energy harvesting module with the overall bearing system.

For electromagnetic generation technology, traditional electromagnetic generator structures are usually large, which limits their application in small bearings. Future designs should prioritize the miniaturization of electromagnetic generator structures while improving their compactness to meet the demands of integrated applications. For piezoelectric generation technology, the key to efficient energy harvesting lies in the close integration of materials and structures. Future piezoelectric generator structural designs could adopt flexible integrated structures, such as embedding piezoelectric films into the rolling elements or cages of bearings to achieve a structural-function integration design. Optimizing the geometric structure of piezoelectric materials, especially combining nano or micron-level designs with materials, can effectively improve energy harvesting efficiency.

The design challenge for thermoelectric generation technology is how to effectively utilize the temperature difference in bearings. Future research could explore integrating micro-thermoelectric modules into the outer or inner rings of bearings, enhancing the contact area between the heat source and thermoelectric materials through precise structural design to improve the heat transfer efficiency. Moreover, the integration of thermoelectric modules should consider the dynamic characteristics of bearings, ensuring stable performance under dynamic conditions.

For triboelectric generation technology, non-contact structures can effectively avoid the wear issues caused by friction contact. Integrating nano-scale structures (such as nano-pillar arrays or microstructured surfaces) can significantly enhance triboelectric performance. Integration with the overall bearing structure should further simplify design and reduce complexity. For tribovoltaic generation technology, independent structures can eliminate the limitations of moving end leads, and integrating semiconductor materials deeply with bearing structures—such as creating semiconductor coatings or directly processing semiconductors into high-strength bearing components—will be key. Additionally, micro-nano structure processing on the surface of semiconductor materials can reduce friction coefficients and improve wear resistance.

### 8.3. Multi-source energy collaborative harvesting

In future self-powered bearing systems, multi-source energy collaborative harvesting will be an important direction for improving energy recovery efficiency and system stability. Different energy harvesting technologies have different working principles and applicable ranges. By combining multiple technologies, not only can the stability of energy harvesting be improved, but it can also effectively address energy demands under different operating conditions, particularly under complex loads and environmental conditions.

Both tribovoltaic and thermoelectric generation technologies are based on semiconductor materials, so their collaborative operation can collect energy from both the mechanical motion of bearings and the heat energy caused by friction or temperature changes. For example, under high load or high-speed rotation conditions, the bearing surface temperature often rises due to friction. At this point, thermoelectric generation technology can effectively harvest friction heat, while tribovoltaic generation can form a potential difference between the friction pairs of the bearing, directly extracting electrical energy from mechanical friction. By combining these two technologies, the system can simultaneously collect energy from both mechanical and thermal sources, significantly improving overall energy recovery efficiency. For example, high-temperature bearings in gas turbines are well-suited for power generation technologies that combine tribovoltaic and thermoelectric effects.

The combination of piezoelectric and triboelectric generation technologies holds great potential. Piezoelectric materials exhibit high sensitivity under high-frequency micro-vibrations, enabling efficient energy conversion during rapid deformation. In contrast, triboelectric technology excels at capturing energy from low-frequency, low-speed motion. The synergistic design of these two technologies can cover a broader range of mechanical motion, significantly enhancing power generation capability. In low-speed bearings, triboelectric technology can continuously output baseline electrical energy through friction generated by the slow rotation of the bearing, while piezoelectric materials can capture high-frequency micro-vibrations (such as structural resonance) occurring during bearing operation. This complementary approach compensates for the energy harvesting limitations of a single mode in specific frequency ranges, ensuring a stable power supply under complex working conditions.

Dynamic impedance matching technology will play an important role in multi-source energy collaborative harvesting, especially when multiple energy harvesting technologies are working in parallel. The output characteristics of different generation technologies vary, and how to balance their outputs will directly impact the system's energy conversion efficiency. By designing power management systems, the impedance matching of each energy harvesting module can be dynamically adjusted to maximize energy conversion efficiency. For example, triboelectric nanogenerators have very high internal resistance, while electromagnetic generators have very low internal resistance. When they are integrated into the bearing system and work together, their electrical outputs need to be managed so that their internal resistance levels are matched, and they can charge capacitors or directly power sensors.

### 8.4. Standardization and low-cost industrialization

To promote the widespread commercialization of self-powered smart bearings, research on standardization and low-cost industrialization pathways is critical. With the continuous advancement of manufacturing processes, low-cost production technologies will become key to driving the industrialization of self-powered smart bearings. For example, the application of 3D printing and screen printing technologies can reduce manufacturing costs. With 3D printing, complex bearing structures can be quickly customized for small batch production, reducing processing time and cost. Additionally, unified standards need to be established for the five energy harvesting technologies to provide consistent technical evaluation and quality assurance for the industry. These standards should cover key performance indicators such as energy conversion efficiency, durability, temperature rise threshold, vibration tolerance, and power output. Standardized testing methods can evaluate voltage output stability and energy conversion efficiency across different speed ranges. For piezoelectric generation technology, standards should clearly define its performance in low-frequency, high-amplitude, or small-amplitude vibrations and its durability. For thermoelectric generation technology, standardized tests should focus on temperature response capability, thermal power output, and thermal loss. For triboelectric and tribovoltaic generation technologies, standardization efforts should focus on surface charge density, power density, and system stability under high-load conditions. Establishing these standards will not only provide direction for technological innovation but also provide quantitative criteria for technology selection in the market, helping industries to reasonably choose the best technological solutions for different application scenarios. Standardized performance testing and certification systems will effectively ensure the product quality of self-powered smart bearings and increase market trust in this emerging technology.

### 8.5. Summary

In conclusion, this review comprehensively discusses the applications and development trends of electromagnetic, piezoelectric, thermoelectric, triboelectric, and tribovoltaic power generation technologies in self-powered smart bearings. Each technology has its own characteristics, with specific working scenarios and limitations: electromagnetic technology is suitable for stable-speed scenarios, piezoelectric technology is sensitive to small vibrations, thermoelectric technology can recover frictional heat, triboelectric technology offers the advantages of low-cost and widely available materials, and tribovoltaic power generation technology, as the latest power generation technology for self-powered bearings, is suitable for in-situ monitoring of high-load bearings. However, challenges remain, especially in terms of material durability, energy conversion efficiency, and system integration. Future smart bearings will trend toward integration and multifunctionality, with materials science, structural design, and intelligent control becoming key research directions. Material innovation and interface optimization are expected to enhance the performance of tribovoltaic and piezoelectric materials, while efficient system integration and multi-source energy collaborative collection will further improve energy recovery efficiency. Meanwhile, with the introduction of intelligent technologies, smart bearings will be able to achieve autonomous diagnosis and predictive maintenance, greatly enhancing the reliability and operational efficiency of equipment. In summary, with the progress of technology, particularly in energy collection efficiency, material optimization, intelligent control, and exploration of industrialization paths, future self-powered smart bearings will play an increasingly important role in industrial Internet of Things (IIoT), intelligent manufacturing, and other fields, driving improvements in mechanical system efficiency and extending equipment lifespan.

## Declaration of competing interest

The authors declare that they have no conflicts of interest in this work.
